# Genome-Wide Identification and Analysis of FKBP Gene Family in Wheat (*Triticum asetivum*)

**DOI:** 10.3390/ijms232314501

**Published:** 2022-11-22

**Authors:** Qiang Ge, Peipei Peng, Mingyue Cheng, Yanjun Meng, Yuan Cao, Shuya Zhang, Yu Long, Gezi Li, Guozhang Kang

**Affiliations:** 1 National Engineering Research Center for Wheat, Henan Agricultural University, Longzi Lake Campus, Zhengzhou 450046, China; 2 National Key Laboratory of Wheat and Maize Crop Science, Henan Agricultural University, Longzi Lake Campus, Zhengzhou 450046, China; 3 State Key Laboratory of Crop Stress Adaptation and Improvement, School of Life Sciences, Henan University, Kaifeng 475004, China

**Keywords:** FKBP, PPIase, qRT-PCR, biotic and abiotic stress

## Abstract

FK506-binding protein (FKBP) genes have been found to play vital roles in plant development and abiotic stress responses. However, limited information is available about this gene family in wheat (*Triticum aestivum* L.). In this study, a total of 64 *FKBP* genes were identified in wheat via a genome-wide analysis involving a homologous search of the latest wheat genome data, which was unevenly distributed in 21 chromosomes, encoded 152 to 649 amino acids with molecular weights ranging from 16 kDa to 72 kDa, and was localized in the chloroplast, cytoplasm, nucleus, mitochondria, peroxisome and endoplasmic reticulum. Based on sequence alignment and phylogenetic analysis, 64 *TaFKBPs* were divided into four different groups or subfamilies, providing evidence of an evolutionary relationship with *Aegilops tauschii*, *Brachypodium distachyon*, *Triticum dicoccoides*, *Arabidopsis thaliana* and *Oryza sativa*. Hormone-related, abiotic stress-related and development-related cis-elements were preferentially presented in promoters of *TaFKBPs*. The expression levels of *TaFKBP* genes were investigated using transcriptome data from the WheatExp database, which exhibited tissue-specific expression patterns. Moreover, *TaFKBPs* responded to drought and heat stress, and nine of them were randomly selected for validation by qRT-PCR. Yeast cells expressing *TaFKBP19-2B-2* or *TaFKBP18-6B* showed increased influence on drought stress, indicating their negative roles in drought tolerance. Collectively, our results provide valuable information about the FKBP gene family in wheat and contribute to further characterization of FKBPs during plant development and abiotic stress responses, especially in drought stress.

## 1. Introduction

Bread wheat (*Triticum asetivum* L.) is an important cereal crop and is cultivated world-wide, feeding more than 35 percent of the global population. Wheat provides more than one-fifth of calorific daily intake around the world, and is also considered a significant source of protein and various minerals [1]. The global population is expected to reach 9.9 billion (2020 Population Reference Bureau) by 2050, requiring increases in wheat production of more than 2 percent per year. Increasing wheat yields while facing and overcoming various biotic and abiotic stresses presents a challenge for scientists.

Multiple proteins, including transcription factors, protein kinases and immunophilins, have been reported to participate in plant abiotic and biotic stress responses, which entails a complicated network [2,3,4]. Immunophilins, as receptor proteins for immunosuppressive drugs, could bind Cyclosporine-A (CsA), FK506 and rapamycin, and are divided into two subfamilies as cyclophilins (cyclosporin A-binding proteins) and FKBPs (FK506/rapamycin-binding proteins) according to its way of binding drugs [5,6]. The FKBP family gene shares a conserved domain of peptidyl-prolyl cis-trans isomerase (PPIase), which could catalyze a proline residue of target protein between cis and trans configurations, associating with protein folding and maturation [7,8]. Based on domain organization, FKBPs were divided into two groups: a single-domain form and a multi-domain form. Single-domain FKBPs have a single FK506-binding domain (FKBD), while multidomain FKBPs have either additional FKBDs with just a tetratricopeptide repeat (TPR) or another functional domain, including C-terminal calmodulin-binding domains or a coiled-coil domain [9,10,11]. FKBPs are widely spread from prokaryotes to eukaryotes, including in *Chlamydomonas*, yeast, plants and humans [8,12,13,14].

In plants, FKBPs play various roles in response to abiotic and biotic stress, including pathogen attacks, heat, cold and drought stresses. AtFKBP15-1, localizing in the endoplasmic reticulum (ER) and possessing PPIase activity, was suppressed by a *Phytophthora capsica* RXLR effector PcAvr3a12, that positively regulates plant immunity in response to Phytophthora infection [15]. In Arabidopsis, AtFKBP65 can induce callose accumulation in the cell wall in response to *Pseudomonas syringe* infection [16]. In addition, FKBP62-FKBP65-HSP90.1 formed a heterotrimer to regulate the activity of the heat shock transcription factor Hsfsa2, which participated in thermotolerance in Arabidopsis [17,18,19]. Meanwhile, ROF1 (rotamase FKBP1), comprising a typical FKBP domain and two additional TPR domains, as well as a calmodulin-binding domain, was found to take part in heat and osmotic/salt stress with partner proteins. NBR1 (next to BRCA1 gene 1) interacted with ROF1 to mediate its degradation during heat stress in Arabidopsis [20]. Moreover, ROF1 interacted with PI(3)P (phosphatidylinositol-3-phosphate) and PI(3,5)P2 (phosphatidylinositol-3,5-bisphosphate) in the osmotic/salt stress responses of germinating *Arabidopsis* seedlings during salt stress [21]. *PaFKBP12* (from *Polytrichastrum alpinum*) was ectopically expressed in *Arabidopsis*, which showed positive responses to heat, salt and drought stresses [22]. Likewise, yeast cells expressing *OsFKBP20* showed improved heat tolerance [23]. In wheat, TaBI-1.1 interacted with TaFKBP62 on the ER membrane, which regulates wheat heat tolerance [24]. Altogether, the FKBP family genes play important roles in various abiotic and biotic stress responses. 

Advances in sequencing technologies have allowed for the sequencing of plant genomes, allowing for the accumulation of large amounts of data. Identified genes in plant genome databases are often not fully characterized, especially in terms of their function and regulation. To better understand the function of those genes, it is important to annotate their characterization, including chromosomal position, gene structure and homology. Gene family analysis provides an effective way to characterize multiple genes concurrently. FKBPs participate in various abiotic stress responses. Based on the highly conserved FK506-binding domain, FKBP genes were analyzed on the whole genome sequence of *Arabidopsis*, rice, maize and apple [24,25,26,27]. To further explore the function of FKBP genes in wheat, 64 FKBPs genes were identified in the wheat genome, and their FK506-binding domain, gene and protein structure, conserved domain, phylogenetic relationships, chromosomal locations, cis-acting elements, protein–protein interactions between FKBPs, the expression pattern in various tissues and their responses to heat, drought, and heat plus drought stress were further examined. Moreover, TaFKBP19-2B-2 and TaFKBP18-6B negatively regulated drought stress when they were ectopically expressed in yeast cells. In our study, we identified 64 genes conserving the FKBP domain in the wheat genome and analyzed their phylogenetic relation, the expression pattern in various tissues, and their response to abiotic stresses.

## 2. Results

### 2.1. Identification of TaFKBP Gene Family in T. aestivum

A total of 64 FKBP genes were identified in the wheat genome by Hidden Markov Model (HMM) profiles of the FKBP domain (PF00254) and confirmed by Conserved Domain Database (CDD) examination, the Simple Modular Architecture Research Tool (SMART) and the Protein Families (PFAM) database to eliminate sequences without FKBP domains (Table 1). This number is relatively higher than previously reported FKBPs in other species such as Arabidopsis, rice, maize and *Chlamydomonas* (Table 2).

For the nomenclature of wheat FKBPs, we considered the evolutionary relationship of *Arabidopsis* and rice FKBP proteins, and used the same suffix number for genes whose orthologs could be classified in the same branch. These include OsFKBP12, -13, -15-1, -15-2, -15-3, -16-1, -16-2, -16-3, -16-4, -17-1, -17-2, -18, -19, -20-1, -20-2, -42a, -53, -62, -62b, -62c, and -72 for FKBPs. The TaFKBPs family had coding sequence (CDS) lengths ranging from 445 to 2508 bp, and varied in molecular weight from 16.02 to 72.08 kDa. TaFKBP62c-2D had the highest molecular weight (72.08), while TaFKBP15-1-5B had the lowest molecular weight (16.02) (Table 1).

The molecular weight of TaFKBPs with their pI was plotted to examine the molecular weight distribution of different TaFKBP family members (Figure S1). The plots show that parts of TaFKBPs with similar molecular weights and pIs are clustered together. Moreover, 35 TaFKBPs are basic (pI > 7), and 29 TaFKBPs are acidic (pI < 7). The calculated grand average of hydropathy index (GRAVY) values of all TaFKBPs were between −1.02 to 0.18, meaning there are 9 hydrophobic and 55 hydrophilic TaFKBPs in nature. Protein subcellular localization is closely related to its function. To better understand TaFKBP function, their subcellular localization was predicted in the CELLO server v2.5 (http://cello.life.nctu.edu.tw/, accessed on 20 October 2021) [28], which showed they were located on different parts of cell organelles, including the nucleus, cytoplasmic, chloroplast, endoplasmic reticulum, mitochondria, peroxisome, and were even secreted (Table 1).

**Figure 1:**
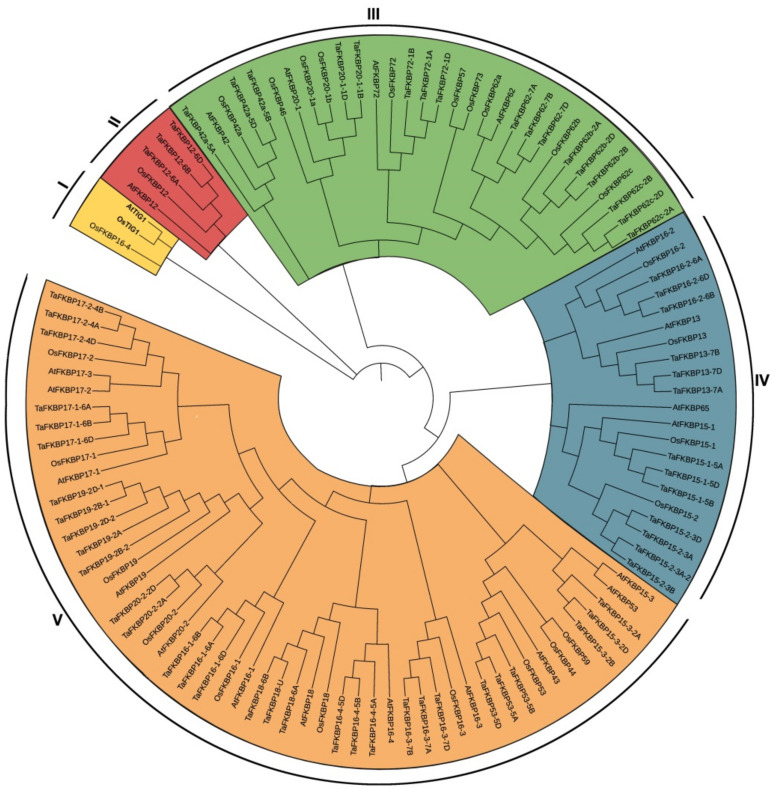
Phylogenetic tree of TaFKBPs. The phylogenetic tree was constructed by using the neighbor-joining method with 1000 bootstrap replications with MEGA X.

To explore the evolutionary relationship among FKBPs in different species, 64 TaFKBPs, 23 AtFKBPs and 29 OsFKBPS were used to construct a phylogenetic tree using MEGA version 10 (Figure 1, Table 2). The results revealed that TaFKBPs were divided into four groups: group V was the largest with thirty members, while groups II, III and IV have 3, 17 and 33 members, respectively.

### 2.2. Chromosomal Distribution of TaFKBP Genes

**Figure 2:**
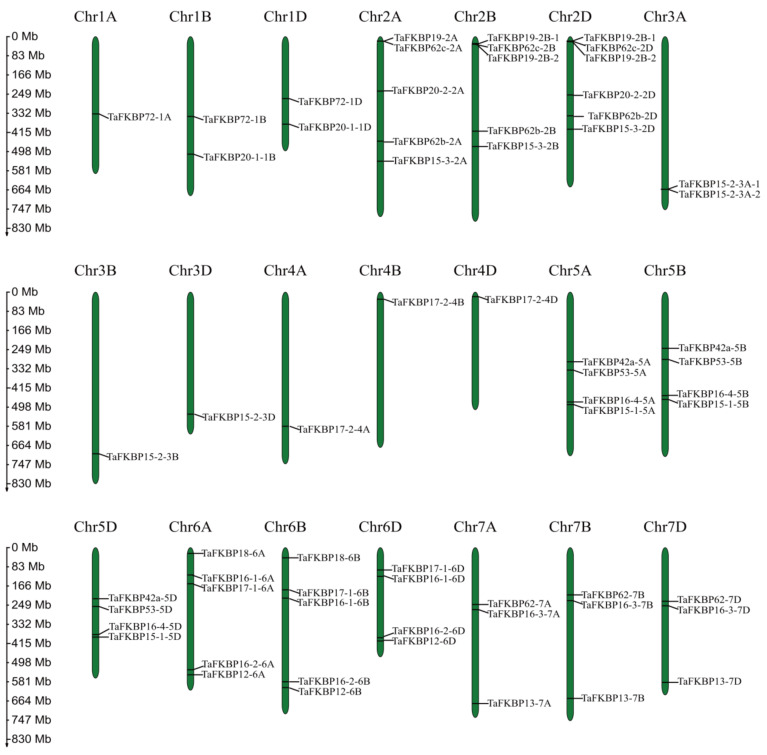
Chromosomal distribution of TaFKBP genes. Each TaFKBP was mapped to its chromosomal position by its physical positions of wheat genomes. The chromosome number is labeled at the top of each chromosome. The scale bar is in mega bases (Mb).

According to the chromosomal locations of FKBP genes per the PhenGram online server, the genomic chromosomal distribution of the identified TaFKBP genes in wheat was mapped onto the corresponding chromosomes. TaFKBP genes are presented on 21 wheat chromosomes (Figure 2). FKBP family genes were unevenly distributed on the three subgenomes of wheat; 27 were on the D subgenome, 24 were on the A subgenome, and 13 were on the B subgenome. Chromosome 6A had the most *TaFKBPs*, with six, while chromosomes 1A, 1B, 2B, 3B, 4B, 5B and 6B had only one *TaFKBP*.

To generate new gene members in the gene family, tandem and segmental duplications constantly occurred. In our data, 56 FKBP genes were found to participate in duplication events, which indicated that the expansion of the FKBP gene family in wheat was caused mainly by whole-genome duplication or segment duplication within genomes (Figure S3). 

**Figure 3:**
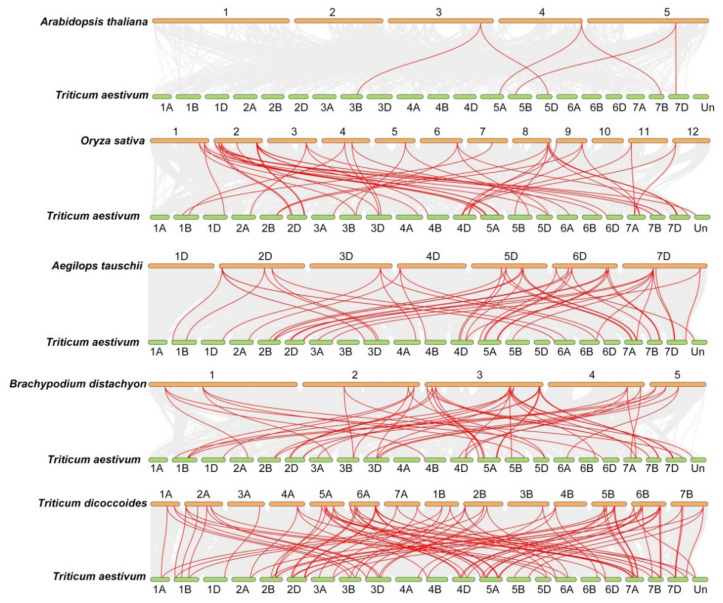
Syntenic relationships of TaFKBP genes between *Aegilops tauschii*, *Brachypodium distachyon*, *Triticum dicoccoides*, *Oryza sativa* and *Arabidopsis thaliana*. The gray lines in the background represent the collinear blocks with *Triticum aestivum* genomes, while red lines highlight the syntenic *FKBP* gene pairs. The labels within the figure indicates chrosomal name.

To further examine the synteny relationships of *TaFKBP* genes with *Aegilops tauschii*, *Brachypodium distachyon*, *Triticum dicoccoides*, *Arabidopsis thaliana* and *Oryza sativa*, a Multiple Collinearity Scan toolkit was used to search the orthologous genes between those genomes (Figure 3). The results showed 44, 48, 84, 5 and 46 orthologous gene pairs between *TaFKBPs* with *AeFKBP*, *BdFKBP*, *TdFKBP AtFKBPs* and *OsFKBPs*, separately. This suggests that TaFKBPs genes could originate from the orthologous genes of an ancestral plant species and expand in wheat.

### 2.3. Gene Structure and Conserved Motif Analysis of TaFKBP Genes

**Figure 4:**
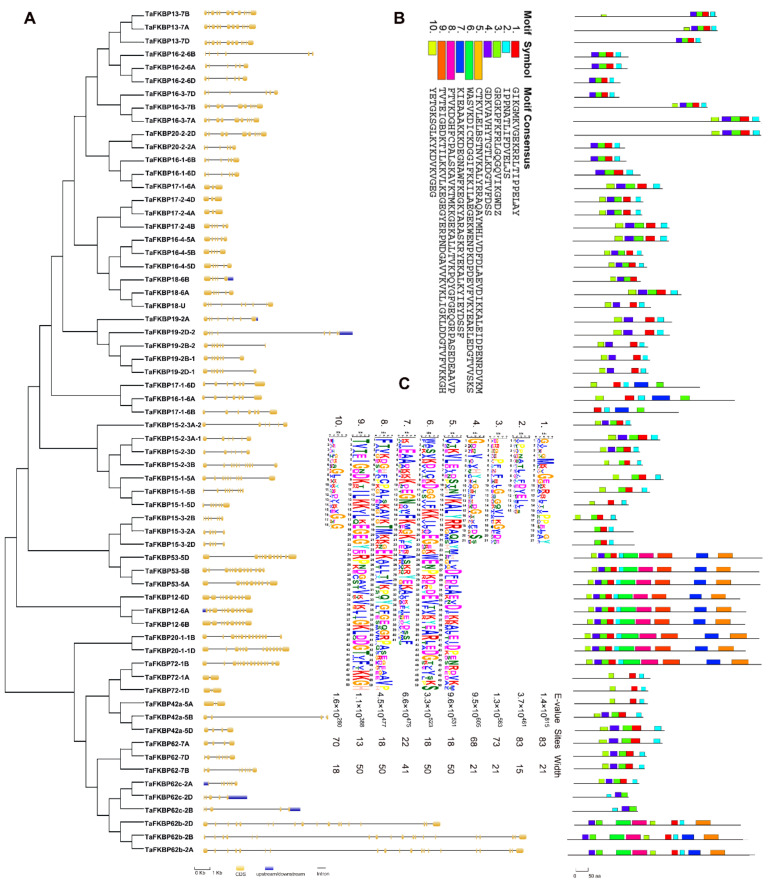
Exon–intron structure and motif distribution of TaFKBP genes. (**A**) Exon–intron structures of TaFKBP genes. Yellow boxes, blue boxes and black lines indicate exons, UTRs and introns, respectively. (**B**) Motif distributions in TaFKBP proteins. The conserved domain of TaFKBP were identified by MEME. Each color indicates a specific domain. (**C**) The conserved motifs of the wheat FKBP protein sequence. The integral height of the stacks indicates the degree of conservation at this site, while each letter in the stacks showed the frequency of the corresponding amino acid.

To explore the structural characteristics of the TaFKBP genes, the exon–intron architectures of TaFKBP genes were analyzed. TaFKBP genes showed large divergences in their number of exons–introns, varying from 1 to 20 (Table S1). Although in the same phylogenetic branch, some pairs of *FKBP* genes such as TaFKBP72-1B/TaFKBP72-1A (19 exons and 20 exons), TaFKBP62c-2A/TaFKBP62c-2D (10 exons and 13 exons) and TaFKBP62-7A/TaFKBP62-7D (13 exons and 15 exons) showed a variable number of exons (Figure 4A).

The conserved motifs of TaFKBP proteins were illustrated using MEME (Multiple Em for Motif Elicitation) online servers. Finally, 10 conserved motifs were identified in 64 TaFKBP proteins (Figure 4B,C). The TaFKBP protein family was identified by the presence of an FKBP-type peptidyl-prolyl cis-trans isomerase domain (Pfam 00254), and all TaFKBPs had at least one FKBP-type peptidyl-prolyl cis-trans isomerase domain (Table S2), which could catalyze a cis/trans interconversion at the proline residue of targeted protein participating in multiple biological processes, including development, abiotic and biotic stress responses. In the TaFKBP protein family, the number of conversed motifs varied from 3 to 10. The TaFKBP protein showed a similar protein structure in the same phylogenetic tree branch, indicating that they share a similar function. 

### 2.4. Cis-Acting Regulatory Elements Analysis of TaFKBP Genes

**Figure 5:**
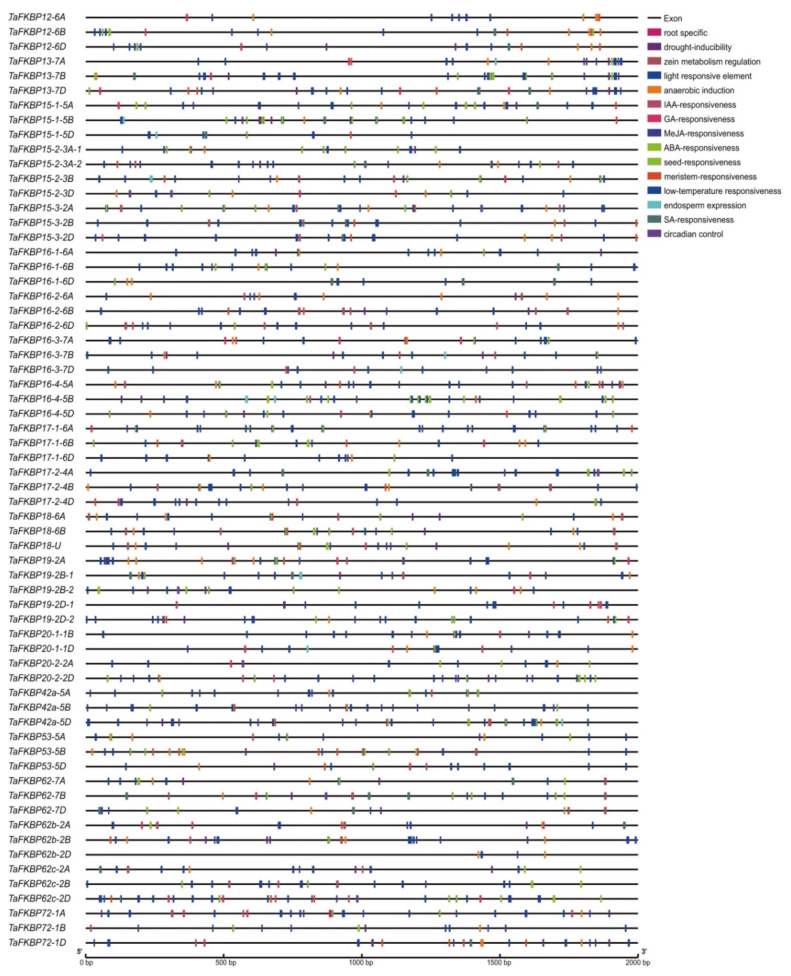
Predicted cis-acting elements in the promoters of *TaFKBP* genes. The promoter sequences (−2000 bp) of 64 TaFKBP genes analysis by PlantCARE.

To further clarify the potential regulatory mechanism of TaFKBP genes, and how these genes are regulated by phytohormone and stress-responsive elements, the PlantCARE webserver was used to detect putative cis-elements in the 2000 bp promoter region of TaFKBPs. In total, 15 unique CAREs were identified in the TaFKBP gene family: these elements are reported to be involved in the auxin response, abscisic acid response, gibberellin response, methyl jasmonate response (MeJA), low-temperature response and drought response (Figure 5). CAREs involved in the MeJA response were prevalent in the TaFKBPs promoters, followed by abscisic acid, drought and low-temperature response, which indicated that TaFKBP genes could participate in plant stress responses (Figure S4).

Light-responsive CAREs were also prevalent in the TaFKBP gene promoters, indicating that TaFKBP genes could be involved in photosynthesis/non-photosynthesis-based light responses and circadian rhythm-mediated light responses. Meanwhile, TaFKBPs also preserved CAREs related to meristem expression, palisade mesophyll cells and zein metabolism. Those CAREs are present in the TaFKBP genes family, indicating that they participated in multiple biological processes regulated by hormones, light and various tissues. Above all, these data provide meaningful insights to explore the TaFKBP gene family in response to phytohormones, stresses and various developmental processes.

### 2.5. Gene Ontology (GO) Enrichment and Protein–Protein Network Analysis of TaFKBP Genes or Proteins

To better understand the functions of TaFKBP family genes by examining their similarity with other’ genes of known function, all TaFKBPs were effectively annotated and assigned GO terms using AgriGO (Figure S5). Meanwhile, eggNOG-Mapper was used to annotate TaFKBPs, which showed similar results to AgriGO (Table S3).

In the biological process category, TaFKBP genes are enriched in the peptidyl-prolyl cis-trans isomerase activity (GO: 0000413), embryo development (GO: 0009793), lateral root development (GO: 0048527), rRNA processing (0006364), isopentenyl diphosphate biosynthetic process (GO: 0019288) and the very long-chain fatty acid biosynthetic process (GO: 0042761) categories (Figure S5A, Table S4). In the cellular component category, TaFKBPs were enriched in the extrinsic component of the lumen side of the plastid thylakoid membrane (GO: 0035450), the NAD(P)H dehydrogenase complex (GO: 0010598), and the nucleolus (GO: 0005730) (Figure S5B). The prediction of subcellular localization was performed by CELLO, and BUSCO (Table 1) also returned similar results. In the molecular function category, peptidyl-prolyl cis-trans isomerase activity (GO: 0003755) was enriched and mainly involved in catalyzing the proline residue of protein conformation transformation (Figure S5C). Meanwhile, FK506 binding (GO: 0005528), phosphatidylinositol-3,5-bisphosphate binding (GO: 0080025), phosphatidylinositol-3-bisphosphate binding (GO: 0032266) and NADH dehydrogenase activity (GO: 0050136) were highly enriched. In addition, the GO term enrichment also indicated multiple roles of TaFKBP genes, including plant organ development, cell differentiation, embryonic pattern specification, unidimensional cell growth, and response to cytokinin and calmodulin binding. Collectively, these results showed that TaFKBP genes play important roles in plant growth and developmental processes.

**Figure 6:**
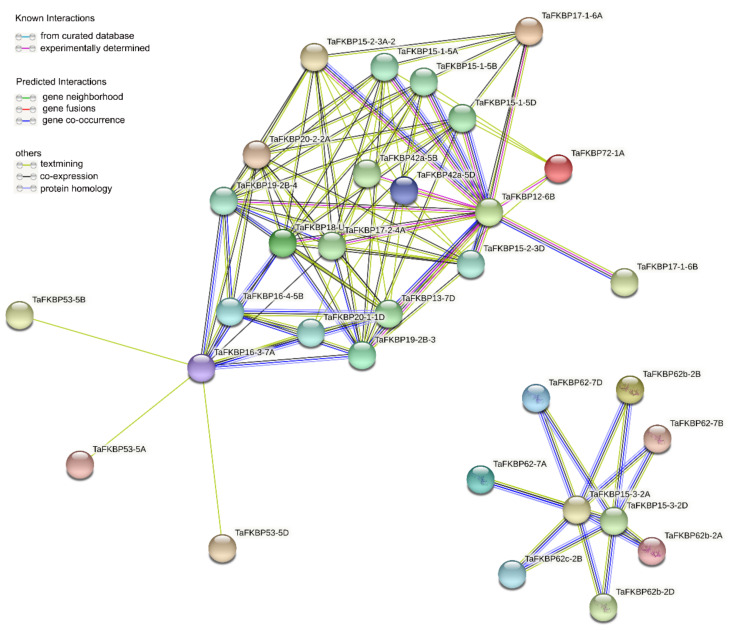
Protein–protein interaction analysis of TaFKBPs protein. Protein–protein interaction network produced by STRINGV9.1. Each node represents a protein, and each edge represents an interaction, colored by the evidence type.

A network was constructed with the STRING database to show the physical (direct) and functional (indirect) associations, which claimed different interactions within studied proteins, showing 32 nodes with an average of 0.58 (Figure 6, Table S5). These results showed 19 representative local network clusters: CL:42766, CL:42743, CL:85687, CL:42740, CL:42737, CL:42738, CL:42736, CL:42734, CL:41959, CL:42733, CL:41962, CL:41972, CL:41966, CL:70404, CL:41965, CL:41969, CL:42796, CL:70406 and CL:49993 (Table S6).

### 2.6. Expression Profiling of TaFKBP Genes under Different Developmental Stages and Stresses

**Figure 7:**
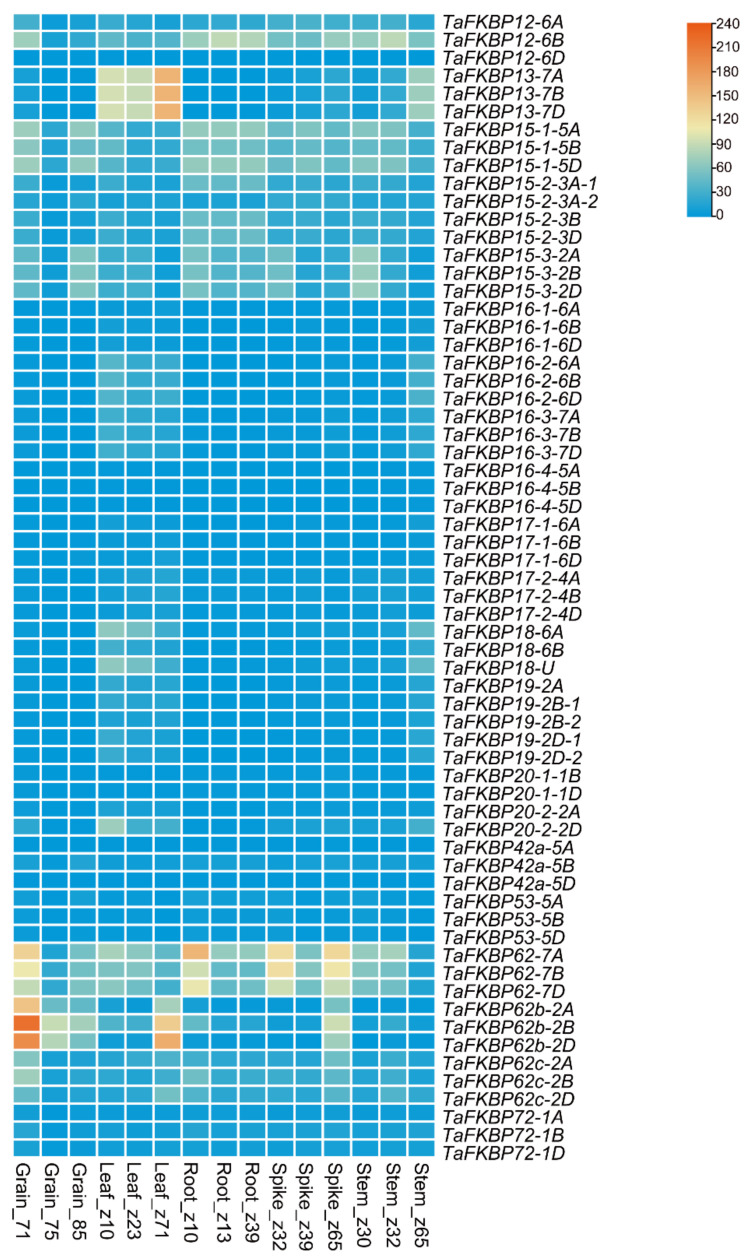
Heatmap representing the expression patterns of TaFKBP genes in various developmental stages. TPM values were directly used to create the heatmap.

The gene expression pattern was closely related to its function. In this study, we investigated the expression level of TaFKBPs in five different tissues (root, stem, leaf, spike and grain) from three different developmental stages retrieved from the WheatExp database. The time points are represented on the Zadoks scale. *TaFKBPs* showed different expression levels in different tissues, even in different development stages. Among the 64 *TaFKBPs*, 6 *TaFKBPs* (*TaFKBP42a-5A/D*, *TaFKBP16-4-5A/B/D*, *TaFKBP12-6D*) were barely expressed in tested tissues. In terms of the gene expression patterns among homologous genes, most homologous genes shared a similar expression pattern, including *TaFKBP12-6*, *-13-7*, -*15-1*, -*15-2*, -*15-3*, -*16-1*, *-16-2*, -*16-3*, -*16-4*, *-17-1*, -*17-2*, -*18-6*, -*19-2*, -*20-1*, -*20-2*, -*53-5*, -*62-7*, -*62b-2* and -*72-1*. However, different expression patterns were observed for *TaFKBP12-6* and *TaFKBP42a* (Figure 7). Totally, these results indicated that different *TaFKBPs* could be involved in the development of different tissues at different stages.

**Figure 8:**
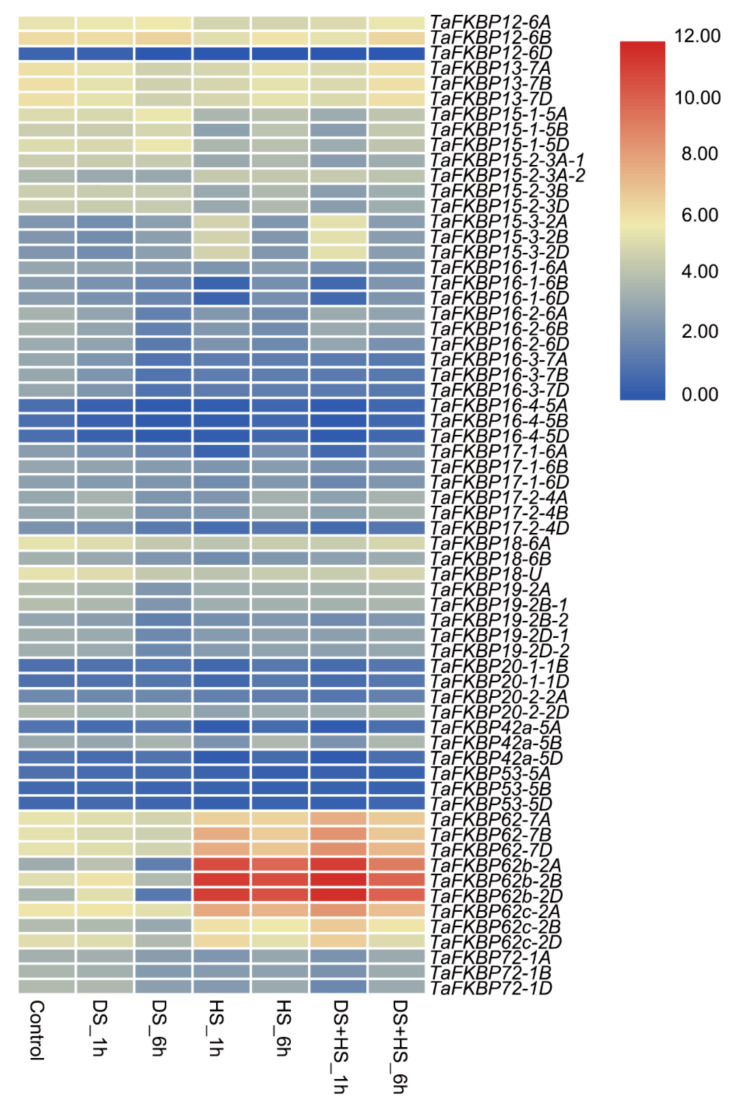
Heatmap representing the expression patterns of TaFKBP genes under heat and drought stresses. TPM values were directly used to create the heatmap. DS: drought stress, HS: heat stress, DS+HS: drought stress plus heat stress.

Global climate change will mean that heat and drought stress will occur more frequently, which will damage crop production. To verify the *TaFKBP* gene’s function under drought and heat stress, we investigated *TaFKBPs* expression trends during drought, heat, and drought plus heat conditions from the WheatExp database. During drought stress, *TaFKBP12-6A/B*, -*15-1-5A/B/D*, -*15-2-3A/B/D*, *-15-3-2A/B/D* and *-42-5B* displayed upregulated expression, while *TaFKBP72-1A/B/D*, *-13-7A/B/D*, *-18-6A/B*, *-18-U*, *-19-2A/B* and -*12-6D* displayed downregulated expression. *TaFKBP62-7A/B/D*, -*62b-2A/B/D* and *-62c-2A/B/D* were induced during heat stress. However, *TaFKBP16-1-6A/B/D*, -*16-2-6A/B/D*, *-16-3-7A/B/D*, *-17-1-6A/B/D*, *17-2-4A/B/D* and *20-2A/D* showed downregulated expression under heat stress. Under heat plus drought stress condition, the expression pattern was similar to that under heat stress. Additionally, *TaFKBP16-4-5A/B/D*, *-20-1B/D*, *-42a-5A/D* and *-53-5A/B/D* slightly varied during drought, heat, and drought plus heat stress (Figure 8). 

**Figure 9:**
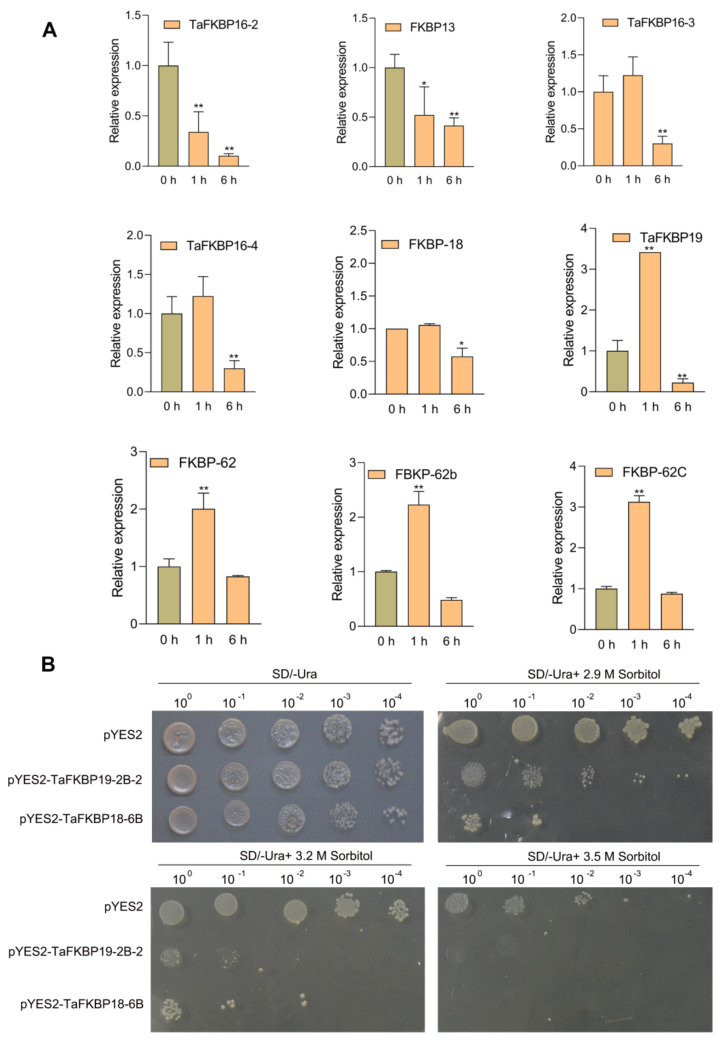
Quantitative real-time PCR analysethe selected TaFKBP genes in response to PEG6000 stress and selected TaFKBPs transformed into a BY4741 yeast cell. (**A**) qRT-PCR analysis of TaFKBP genes after 1 and 6 h under PEG6000 treatment. (**B**) Growth of transgenic yeast cells on SD/−Ura media containing 2.9 M, 3.2 M and 3.5 M sorbitol. The plasmid of pYES2, pYES-TaFKBP19-2B-2 and pYES-TaFKBP18-6B was transformed into yeast strain BY4741, cultured at 30 °C in an incubator for three days. Then, a single positive colony was cultured in SD/−Ura liquid medium until OD600 reached 1, which was diluted and dropped on an SD/−Ura plate containing 0, 2.9 M, 3.2 M and 3.5 M sorbitol. Values are mean ± SD of three biological replicates. Student’s *t*-test was used to estimate the difference. *p* < 0.05 was considered statistically significant (*) and *p* < 0.01 was considered exceedingly statistically significant (**).

Subsequently, the expression level of nine randomly selected *TaFKBP* genes were verified by qRT-PCR under PEG6000 treatment to mimic drought stress. The qRT-PCR result showed that nine selected genes had similar expression patterns with RNA-seq data (Figure 9A). To further confirm its function in drought stress, TaFKBP19-2B-2 and TaFKBP18-6B were chosen to transform into yeast cells, and the transformant showed more sensitivity to drought stress, indicating that these two proteins play negative roles in drought tolerance (Figure 9B). TaFKBP genes responded to heat and drought stress. 

## 3. Discussion

The FKBP family is broadly present in prokaryotes and eukaryotes, and catalyzes the cis-trans isomerization of the prolyl-peptide bond, accelerating the folding of newly synthesized proteins [7,29]. In recent years, FKBPs have been found to participate in organogenesis, seed germination, morphogenesis, and various abiotic or biotic stress responses [22,30,31]. The function of the *FKBP* gene family has been well studied in maize, strawberries, peaches and tomatoes, but not in wheat [32,33,34,35]. 

Genome-wide analysis of a gene family is a way of rapidly and efficiently analyzing pathways to characterize gene functions and evolution [36,37,38,39]. In wheat, various genes are involved in drought, salt and metal stress responses by characterizing genes families [40,41,42]. In this study, 64 FKBPs were identified in the wheat genome by systematic in silico analysis (Table 1). Phylogenetic analysis showed that TaFKBPs, OsFKBPs and AtFKBPs were divided into five subgroups or subfamilies (I–V), while TaFKBPs were exclusively presented in groups II to V, but not in group I (Figure 1). AtTIG1 (chloroplast-localized trigger factor1) shared approximately 58 percent of its identities with OsTIG1, eother than any TaFKBPs, which indicated that TIG1 plays a special role in *Arabidopsis* and rice [43,44]. Only FKBP12 in group II showed its typical functions participating in physiological and morphological establishment, such as flowering and root formation [45,46]. In Group V, wheat had approximately three times as many FKBP genes as *Arabidopsis* and rice counterparts, showing that extensive duplication of these TaFKBPs occurred during evolution. Moreover, most TaFKBPs were well allocated into the known group of *Arabidopsis* and rice. 

Gene duplication is the main driving force of the expansion of gene families in different species, which mainly involves segmental, tandem and whole-genome duplication [47,48]. Compared with *Arabidopsis* (23), rice (29), maize, (30) and *Chlamydomona* (23) FKBP gene families, the wheat genome contained more FKBPs and was distributed on 21 chromosomes, varying from one to six FKBPs on different chromosomes. The chromosomes 1A, 1B, 2B, 3B, 4B and 5B have a single FKBP, while chromosomes 2A, 3A, and 7A have two FKBPs, chromosomes 5C, 6C and 7C have three FKBPs, chromosomes 6B, 7B, 1C and 4C have four FKBPs, chromosomes 4A, 5A, 2C and 3C have five FKBPs, and chromosome 6 has six FKBPs. Among 64 TaFKBPs, 23, 19 and 27 TaFKBPs were found on the A, B and D sub-genomes, respectively, which indicated that gene loss could be present in the wheat FKBP gene family, resulting in the loss of some homologous copies. When analyzing the synteny relationships of TaFKBPs with *B. distachyon*, *Ae. tauschii*, *T. dicoccoides*, *O. sativa* and *A. thaliana*, we identified 48, 44, 84, 46 and 5 orthologous gene pairs, respectively (Figure 3). The results indicated that TaFKBPs had a closer evolutionary relationship with monocots than dicots. 

Gene structure analysis provided important clues to clarify gene function and evolution. The TaFKBP gene family shared the conserved domain of FKBP-type peptidyl-prolyl cis-trans isomerase, yet the exon-intron architectures varied among 64 TaFKBPs, even in the same group. Group II contained 4 to 6 exons and 3 to 5 introns, while group III contained 5 to 20 exons and 4 to 19 introns. Group IV contained 3 to 6 exons and 2 to 5 introns, and group V contained 2 to 12 exons and 1 to 11 introns. The homology genes always shared a similar exon–intron pattern, while some of the TaFKBPs presented a different exon–intron pattern, similar to TaFKBP16-1-6A (4 exons and 3 introns), TaFKBP16-1-6B (8 exons and 7 introns), TaFKBP16-1-6D (7 exons and 6 introns), TaFKBP17-1-6A (8 exons and 7 introns), TaFKBP17-1-6B (4 exons and 3 introns) and TaFKBP17-1-6D (6 exons and 5 introns). Intron number and size were closely related to gene function, which presented the loss or gain of introns during evolution of the plant due to selection pressures. In addition, genes showed various exon–intron structures presenting diverse functions. The results indicated that gene differentiation in the TaFKBPs family could result in various functions due to selection pressure during wheat genome evolution. 

Motif analysis revealed 10 conserved motifs to illustrate structure comparisons among TaFKBPs proteins, and motif 2 was found in all TaFKBPs proteins identified in this study (Figure 4B). Motif 2 consisted of the FKBP_C domain (Pfam00254), while motifs 5 and 3–4 were part of the tetratricopeptide repeat (TPR, Pfam00515) and the nucleoplasmin-like domain (NPL, Pfam17800), respectively. The TPR motif is a protein–protein interaction module, facilitating specific interactions with a partner protein. NPL is as histone chaperone containing a pentameric N-terminal domain and an unstructured C-terminal tail. Most parts of TaFKBPs contained single or multiple PPI domains, while some parts of TaFKBPs had NPL or TPR domains (Table S2). All the NPL-contained TaFKBP proteins belonged to group V, while five out of six TPR-containing TaFKBP proteins were part of group III, excluding *TaFKBP15-3-2D* belonging to group V. The data showed that TaFKBPs could have conserved and redundant functions. In *Arabidopsis*, a single mutant of AtFKBP15-1 and AtFKBP15-2 did not show any phenotype, while the double mutant *fkbp15-1*/*fkbp15-2* exhibited more lateral roots than the wild type [49]. Meanwhile, three TaFKBP15-1 and four TaFKBP15-2 were found in the wheat genome, showing the highly functional redundancy of FKBP genes. All FKBP12 in rice, wheat and *Arabidopsis* were down-regulated in response to biotic and abiotic stresses [50,51,52]. This indicates that the same group of genes in phylogenetic tree analysis could share similar functions, even in different species.

The subcellular localization of genes had a tight connection with its functions. Nearly half of the TaFKBP genes were predicted to be located in the chloroplast, which shared a similar pattern with AtFKBPs [27]. The chloroplast is a unique plant organelle for photosynthesis and synthesis of a diversity metabolites, which also serve as stress sensors to initiate plastid-to-nucleus retrograde signaling [49]. FKBP16-1, located in the chloroplast, was found to play a role in chloroplast biogenesis in *Arabidopsis* and wheat, and responded to photosynthetic stress [53]. The data suggested that FKBP genes could share a similar function in wheat.

The cis-acting regulatory element is a non-coding DNA sequence distributed in the promoter regions of the gene, which could reveal its regulation and function. In this study, the identified CAREs elements in TaFKBP genes were mainly classified into three categories: phytohormone response, stress response, and growth and development, which contained at least 10 CAREs elements in the promoter region of each TaFKBP gene. Abscisic acid response element (ABREs) elements were presented in all TaFKBP promoter regions. Meanwhile, the TCA-element (salicylic acid responsiveness), P-box and TATC-box (gibberellin-responsive element), CGTCA-motif (MeJA-responsiveness), and the TGA-element and AuxRR-core (auxin-responsive element) were detected in the promoter of TaFKBP genes. Subsequently, five cis-elements were related to growth and development including motif I (root-specific element), the Y-element (seed-specific regulation), the CAAAGATATC-motif (circadian control), the GCN4-motif (endosperm expression), and the O_2_-site (zein metabolism regulation). Moreover, other cis-elements have been implicated in diverse stress conditions, including LTR (low-temperature responsiveness), MBS (drought induction) and ARE (anaerobic induction element). The different numbers and species of cis-elements in the promoter region of the gene had a tight connection with its function. In a previous study, FKBP genes, possessing various numbers and species of cis-elements, showed differential expression under ABA, GA_3_ and MeJA treatment in tomatoes [32]. In wheat, FKBP73 possessed three ABREs elements in the promoter, and were induced 1.9-fold under ABA treatment [54]. In the TaMIOX promoter region, multiple abiotic stress-responsive cis-elements were identified by in silico analysis, which showed significant up-regulation in response to heat (5-fold), cold (7-fold) and drought (5-fold) stress [55]. These results indicate that TaFKBP genes could be induced by a diversity of hormones, stress and developmental processes. The results should be confirmed by further experiments, which will provide valuable clues for understanding how the TaFKBP gene family responds to phytohormones, stresses and developmental processes. Meanwhile, the cis-elements in the promoter region could be a potential target for genome editing to better understand its function, which could provide valuable genes resources for molecular breeding.

In a recent study, FKBP had been found to interact with partner proteins regulating development and stress responses. In Arabidopsis and *Chlamydomonas*, FKBP12 interacted with CO (CONSTANS) to affect its stability regulating flowering [45]. AtFKBP15-1 and AtFKBP15-2 interacted with vacuolar invertase VIN2 to regulate lateral root development [49]. AtFKBP42 was reported to activate ABCB1,19-mediated auxin transport for cell elongation of the stamen [56,57]. In our results, TaFKBP42 was predicted to interact with TaFKBP15-1, -15-2 and -12, which could indicate that TaFKBP42 is involved in auxin signaling, regulating cell elongation (Figure 6). These results provide a valuable clue for identifying the potential biological functions of TaFKBP. 

The expression pattern of a gene is closely related to its function. When analyzing the expression pattern of FKBP genes in various tissues, most TaFKBPs were predominantly expressed in one tissue. TaFKBP15-1 and -15-2 were highly expressed in roots, indicating a potential role in root development (Figure 7). Meanwhile, both of them belong to group IV in the phylogenetic tree (Figure 1). This could indicate that TaFKBP15-1 and -15-2 have similar functions. In a previous study, AtFKBP15-1 and -15-2 were prominently expressed in the roots and worked together to regulate root development [49]. Moreover, homologous genes possessed a similar expression pattern (Figure 7). However, TaFKBP12-6B presented a different expression profile with TaFKBP12-6A/D, which could indicate that these genes had undergone sub-functionalization or neo-functionalization during the wheat evolutionary process. The temporal and spatial expression pattern of TaFKBP genes indicates that these FKBPs could have a function in different tissues and various developmental stages in wheat.

The FKBP gene family plays a vital role in response to abiotic and biotic stresses. In rice, FKBP64, FKBP65 and FKBP75, possessing three FKBP12-like domains and a TPR domain, were induced under heat treatment, which indicated their function in response to heat stress [10]. ROF2, encoding a peptidyl-prolyl cis-trans isomerase, activated K (+) uptake and the electrogenic H (+) pump, which conferred tolerance to intracellular acidification by increasing proton extrusion from cells [58]. FKBP5, as a co-chaperone, modulates glucocorticoid receptor activity in response to stressors [59]. AtFKBP12 mediated the interaction between rapamycin and *Botrytis cinerea* TOR (BcTOR) involved in plant resistance to grey mold [52]. Our results demonstrated that three FKBP genes (TaFKBP62, -62b, -62c, -15-3) were induced under heat treatment, while eight FKBP genes (TaFKBP13, -15-1, -15-2, -16-1, -16-2, -16-3, -16-4, -18-6) were decreased. TaFKBP19-2 decreased its expression in response to drought stress, while the rest of the FKBP genes showed slight changes during drought or heat stress (Figure 8). The expression pattern of TaFKBP genes under drought and heat stress indicated that they could participate in stress tolerance in wheat by coordinating regulation among the TaFKBPs. Moreover, expression profiling of nine randomly selected TaFKBPs was further validated by qRT-PCR. Moreover, qRT-PCR results also exhibited a similar expression with slight variation (Figure 9A). Among the selected genes, TaFKBP18 and TaFKBP19 were highly expressed in the leaves and had decreased expression under drought stress, indicating that both of them negatively affected drought tolerance. When TaFKBP18-6B and TaFKBP19-2B-2 were ectopically expressed in yeast cells, showing a lower expression between homologous genes, their drought tolerance decreased (Figure 9B). These results provide vital clues for clarifying *TaFKBP* function in response to abiotic stresses.

In total, we identified 64 TaFKBP genes in the wheat genome, and analyzed their gene structure, phylogenetic relationships, chromosomal locations, cis-acting elements, protein–protein interaction network and expression patterns in various tissues or under drought and heat stress. Our work provides important information for further elucidating the role of the TaFKBP gene family in plant growth and development or under heat and drought treatment.

## 4. Materials and Methods

### 4.1. Identification of FKBP Genes in the Wheat Genome

The genome sequence data and the annotation information of wheat (Chinese spring) were obtained from the Ensembl Plants database (http://plant.ensembl.org/index.html accessed on 9 October 2021). FKBP protein data of *Arabidopsis thaliana* (At) and *Oryza sativa* (Os) were downloaded from TIAR (http://www.Arabidopsis.org/ accessed on 9 October 2021) and Rice Genome Annotation Project (http://rice.uga.edu/ accessed on 9 October 2021). The hidden Markov models (HMM) of FKBP protein (Pfam accessions: PF00254) were downloaded from the Pfam database (http://pfam.xfam.org/ accessed on 15 October 2021) and were used as queries to search for potential FKBP proteins in the wheat protein datasets by using HMMER3.0 with an E-value cutoff of 10^−5^ [60]. Based on the above method, putative candidate genes were selected. In addition, candidate protein sequences of FKBPs were subjected to online domain analysis program NCBI–CDD (https://www.ncbi.nlm.nih.gov/cdd/ accessed on 15 October 2021), and SMART (http://smart.emblheidelberg.de/ accessed on 15 October 2021) to confirm the presence of the conserved domain in the predicted TaFKBP proteins [61,62]. Finally, 64 protein sequences with FKBP-type peptidyl-prolyl cis-trans isomerase domains were taken and named sequentially according to its homology with corresponding MAPKK proteins of Arabidopsis or rice and their locations on the wheat chromosomes.

### 4.2. Physico-Chemical Characteristics, Subcellular Localization, Gene Structure, Multiple Sequence Alignment and Construction of Phylogenetic Tree

The protein characteristics, including isoelectric point, lengths and molecular weight of TaFKBP proteins, were evaluated by isoelectric point calculator and ExPASy (https://web.expasy.org/compute_pi/ accessed on 20 October 2021). Subcellular localization was predicted using CELLO (http://cello.life.nctu.edu.tw/ accessed on 20 October 2021) and the WoLEPSORT online tool (http://www.genscript.com/wolf-psort.html accessed on 20 October 2021) [28,63,64,65,66].

The exon/intron structures were constructed by GSDS (http://gsds.gao-lab.org accessed on 20 October 2021) using the coding sequences (CDS) and corresponding genomic sequences retrieved from the Ensembl Plants database2 [67]. Multi-sequence alignments were carried out using the ClustalW version 2.0 with default settings. Phylogenetic and molecular evolutionary analysis was conducted by MEGA version X using the neighbor-joining and maximum likelihood methods. The reliability of phylogenetic trees was tested using bootstrapping with 1000 replicates [68].

### 4.3. Chromosome Localization, Motif Analysis and Gene Ontology

For the distribution on chromosomes, genomic positions of FKBP genes were downloaded from the Ensembl Plants BioMart (http://plants.ensembl.org/biomart/martview accessed on 30 October 2021). MapGene2Chrom (http://mg2c.iask.in/mg2c_v2.1/ accessed on 30 October 2021) was used to represent TaFKBP genes on the wheat chromosomes [69]. The MEME tool (http://meme-suite.org/tools/meme accessed on 30 October 2021) was used to elucidate TaFKBP conserved motifs with the following parameters: optimum motif width set to ≥6 and ≤50; number of motifs: 10. To explore gene ontology, Gene Tribe (https://chenym1.github.io/genetribe/ accessed on 30 October 2021) and EggNOG (http://eggnogdb.embl.de/#/app/emapper accessed on 30 October 2021) were used to predict gene ontology terms with TaFKBPs protein sequences [70,71,72].

### 4.4. Cis-Acting Regulatory Elements (CAREs) Analysis and Protein Interaction Network

To identify CAREs, 2000 bp upstream sequences of FKBP genes were downloaded from Ensemble Plants and analyzed with the PlantCARE online server (http://bioinformatics.psb.ugent.be/webtools/plantcare/html accessed on 20 October 2021) [73,74]. Subsequently, the most commonly occurring CAREs were represented in TBtools [75]. With the STRING online server (https://string-db.org/cgi accessed on 20 October 2021) with the following parameters of required score (0.400) and FDR stringency (5 percent), the TaFKBP protein interaction network was examined [76]. 

### 4.5. Expression Profiling of TaFKBP Genes

Gene expression data of *TaFKBP* genes in different wheat tissues (root, stem, leaf, spike, grain) and various stresses (drought, heat, drought plus heat) of Chinese Spring were obtained from the WheatExp database (http://www.wheat-expression.com accessed on 20 October 2021) [77]. The expression pattern was presented as a heatmap based on FPKM (fragments per kilobase of transcript per million mapped reads), which was mapped by TBtools.

### 4.6. Plant Material and Growth Conditions

Seeds of var. Bainong207, a winter wheat variety mainly cultivated in the Henan province, were grown under controlled glass-house conditions. Fifteen-day-old wheat seedings were subjected to drought (20% PEG6000) and high-temperature stress (37 °C) for 1 h and 6 h. All the samples were immediately frozen in liquid nitrogen and stored at −80 °C for further RNA extraction.

### 4.7. RNA Isolation and Real-Time PCR

Total RNA was extracted with the Applied Biosystems kit (A33784) according to the manufacturer’s protocol. Then, 2 μg isolated RNA was treated with DNase I (TaKaRa, Osaka, Japan) and cDNA synthesis was conducted using the High capacity cDNA kit (Applied Biosystems, 4368813).

Quantitative real-time PCR (qRT-PCR) was performed using the ABI Q5 Real-Time PCR (Applied Biosystems, Waltham, MA, USA). Each qRT-PCR reaction was carried out with three technical replicates and repeated three times. The fold change was calculated based on mean 2^−ΔΔ*CT*^ values and was used for plotting graphs. Wheat actin (AB181991) was used as the internal control to normalize the data [78]. Primer pairs were designed with Primer 5.

### 4.8. Yeast Transformation

The TaFKBP19-2B-2 and TaFKBP18-6B coding sequences were inserted into the pYES2 vector. The vectors pYES2, pYES2-TaFKBP19-2B-2 and pYES2-TaFKBP18-6B were transformed into yeast strain BY4741 as previously described [79]. After culturing on SD agar medium lacking Ura at 30 °C for 3 days, yeast transformants were used for making series of dilutions (10^−1^) and 8 μL samples of each diluted culture were plated onto SD/-Ura plates containing 0, 2.9 M, 3.2 M and 3.5 M sorbitol as described.

### 4.9. Statistical Analyses

Each experiment was performed in triplicate. The present data were analyzed after calculating the mean ± standard deviation (SD) of each experiment with SPSS22. Student’s *t*-test was used to estimate the difference; *p* < 0.05 was considered statistically significant and *p* < 0.01 was considered exceedingly statistically significant.

## 5. Conclusions

Bread wheat is an important cereal crop, grown worldwide, and is a staple food for more than 20 percent of the global population. As a result, many scientists around the world are attempting to improve yield by addressing various environmental stresses of wheat. In past decades, the FKBP gene family has been found to participate in plant growth and stress responses. In our study, we identified 48 TaFKBPs containing a conserved FKBP domain in the wheat genome, as well as the gene position, subcellular localization, isoelectric point, molecular weight, phylogenetic relationships, gene and protein structure, and cis-elements. Expression profiling was also identified to show its potential roles in various developmental stages and stress conditions, which indicated the TaFKBPs involved in drought stress. Furthermore, ectopic expression of TaFKBP19-2B-2 and TaFKBP18-6B in yeast cells negatively regulated drought stress. Therefore, our study provides candidate genes for improving plant growth and stress tolerance in bread wheat, which contributes to a better understanding of *TaFKBP* function in the various developmental stages and stress responses of wheat.

## Figures and Tables

**Table 1:** Nomenclature and characteristics of the putative FKBP proteins in wheat.

Name	Gene ID	GRAVY	MW	Start	End	Chromosome	CDS Length (bp)	Protein Length (aa)	PI	Localization
TaFKBP72-1A	TraesCS1A02G184600	0.55	69.61	335,012,317	335,034,601	1A	2332	624	5.75	Nuclear, Cytoplasmic
TaFKBP72-1B	TraesCS1B02G192700	0.48	67.97	345,303,083	345,325,533	1B	2055	607	5.52	Chloroplast, Mitochondrial
TaFKBP20-1-1B	TraesCS1B02G292400	0.42	21.78	509,891,166	509,892,675	1B	609	202	7.35	Nuclear, Cytoplasmic
TaFKBP72-1D	TraesCS1D02G192100	0.51	63.98	267,788,383	267,804,883	1D	2195	575	5.26	Nuclear, Cytoplasmic
TaFKBP20-1-1D	TraesCS1D02G282500	0.41	21.74	379,796,335	379,797,841	1D	609	202	7.35	Nuclear, Cytoplasmic
TaFKBP62c-2A	TraesCS2A02G050600	0.52	71.81	19,645,993	19,652,506	2A	2508	645	5.08	Cytoplasmic, Endoplasmic reticulum
TaFKBP19-2A	TraesCS2A02G053200	0.49	29.04	21,261,065	21,267,439	2A	1184	263	10.48	Mitochondrial
TaFKBP20-2-2A	TraesCS2A02G226800	0.40	27.14	236,827,327	236,834,045	2A	735	244	9.88	Chloroplast, Mitochondrial
TaFKBP62b-2A	TraesCS2A02G277100	0.67	64.9	45,805,0841	45,8054,214	2A	2289	582	4.94	Cytoplasmic, Nuclear
TaFKBP15-3-2A	TraesCS2A02G314800	0.97	48.84	539,964,818	539,968,229	2A	1829	444	6.23	Nuclear
TaFKBP62c-2B	TraesCS2B02G063900	0.52	71.31	30,494,467	30,499,674	2B	2404	638	5.24	Cytoplasmic, Endoplasmic reticulum
TaFKBP19-2B-1	TraesCS2B02G067100	0.36	24.28	33,848,383	33,851,216	2B	663	220	9.59	Chloroplast
TaFKBP19-2B-2	TraesCS2B02G067200	0.48	16.83	33,907,018	33,908,399	2B	506	151	5.31	Cytoplasmic, Nuclear
TaFKBP62b-2B	TraesCS2B02G294500	0.69	65.61	411,140,309	411,143,734	2B	2382	587	4.82	Nuclear, Cytoplasmic
TaFKBP15-3-2B	TraesCS2B02G333300	1.00	53.47	476,557,369	476,560,999	2B	1960	487	5.52	Nuclear
TaFKBP62c-2D	TraesCS2D02G050300	0.51	72.08	18,642,151	18,646,485	2D	2174	649	4.99	Endoplasmic reticulum, Cytoplasmic
TaFKBP19-2D-1	TraesCS2D02G053100	0.40	20.91	20,661,350	20,663,246	2D	922	187	8.70	Cytoplasmic, Nuclear
TaFKBP19-2D-2	TraesCS2D02G053400	0.46	29.09	20,778,047	20,783,120	2D	1307	264	10.40	Mitochondrial, Chloroplast
TaFKBP20-2-2D	TraesCS2D02G239700	0.44	21.03	255,681,859	255,683,584	2D	579	192	10.52	Chloroplast
TaFKBP62b-2D	TraesCS2D02G276000	0.65	65.45	345,623,668	345,626,945	2D	2095	550	6.59	Cytoplasmic, Nuclear
TaFKBP15-3-2D	TraesCS2D02G313000	1.02	53.45	402,709,856	402,713,456	2D	2102	487	5.28	Nuclear
TaFKBP15-2-3A-1	TraesCS3A02G421300	0.32	17.53	66,281,3307	66,282,0850	3A	471	156	5.78	Secreted
TaFKBP15-2-3A-2	TraesCS3A02G422000	0.19	16.69	663,292,556	663,297,701	3A	728	158	9.10	Secreted
TaFKBP15-2-3B	TraesCS3B02G457500	0.18	16.72	699,693,748	699,696,737	3B	792	158	9.46	Cytoplasmic, Mitochondrial,
TaFKBP15-2-3D	TraesCS3D02G417400	0.18	16.52	529,138,315	529,141,328	3D	786	156	8.47	Secreted
TaFKBP17-2-4A	TraesCS4A02G270400	0.31	26.4	581,749,445	581,750,928	4A	1318	251	5.12	Chloroplast
TaFKBP17-2-4B	TraesCS4B02G043700	0.32	26.76	31,849,101	31,850,336	4B	1092	255	5.40	Chloroplast
TaFKBP17-2-4D	TraesCS4D02G041200	0.29	26.25	19,398,540	19,399,673	4D	1038	250	6.02	Chloroplast
TaFKBP42a-5A	TraesCS5A02G134500	0.58	36.28	303,282,052	303,287,233	5A	1414	315	9.97	Chloroplast
TaFKBP53-5A	TraesCS5A02G158900	0.90	47.59	338,893,087	338,896,927	5A	1810	432	5.24	Nuclear
TaFKBP16-4-5A	TraesCS5A02G264500	0.06	23.29	476,682,552	476,687,413	5A	1069	223	10.66	Chloroplast
TaFKBP15-1-5A	TraesCS5A02G279500	0.15	16.14	488,408,951	488,411,182	5A	665	152	5.78	Secreted
TaFKBP42a-5B	TraesCS5B02G131200	0.59	42.11	243,916,316	243,920,638	5B	1624	370	6.61	Cytoplasmic
TaFKBP53-5B	TraesCS5B02G156700	0.89	47.76	289,160,446	289,164,785	5B	1796	433	5.09	Nuclear
TaFKBP16-4-5B	TraesCS5B02G263800	0.11	23.43	448,220,159	448,223,932	5B	1053	223	10.75	Chloroplast
TaFKBP15-1-5B	TraesCS5B02G278800	0.12	16.02	464,587,956	464,590,390	5B	865	152	6.26	Cytoplasmic
TaFKBP42a-5D	TraesCS5D02G139000	0.58	41.93	221,310,848	221,315,012	5D	1473	370	6.27	Nuclear
TaFKBP53-5D	TraesCS5D02G164000	0.92	47.81	255,575,313	255,579,380	5D	1835	433	5.35	Nuclear
TaFKBP16-4-5D	TraesCS5D02G272100	0.05	23.24	375,377,111	375,387,488	5D	1141	222	10.85	Chloroplast
TaFKBP15-1-5D	TraesCS5D02G286500	0.11	16.06	386,732,844	386,735,285	5D	787	152	5.78	Secreted
TaFKBP18-6A	TraesCS6A02G050000	0.33	24.32	25,636,074	25,636,973	6A	445	138	10.06	Chloroplast
TaFKBP16-1-6A	TraesCS6A02G143200	0.03	21.86	119,619,077	119,621,219	6A	903	207	10.06	Chloroplast
TaFKBP17-1-6A	TraesCS6A02G162000	0.20	23.24	157,134,336	157,138,036	6A	891	212	9.01	Chloroplast
TaFKBP16-2-6A	TraesCS6A02G302500	0.11	22.46	535,990,059	535,991,654	6A	1002	222	9.25	Chloroplast
TaFKBP12-6A	TraesCS6A02G314100	0.23	20.08	550,732,566	550,738,491	6A	864	193	8.94	Chloroplast
TaFKBP18-6B	TraesCS6B02G066300	0.34	24.02	44,104,775	44,108,505	6B	977	225	10.06	Chloroplast
TaFKBP17-1-6B	TraesCS6B02G171400	0.09	21.89	183,753,733	183,762,420	6B	624	207	10.26	Chloroplast
TaFKBP16-1-6B	TraesCS6B02G189300	0.17	23.08	219,095,831	219,097,981	6B	941	212	9.01	Chloroplast
TaFKBP16-2-6B	TraesCS6B02G331700	0.18	22.47	583,050,670	583,052,365	6B	943	221	9.25	Chloroplast
TaFKBP12-6B	TraesCS6B02G344100	0.33	20.49	606,285,921	606,289,161	6B	731	195	9.79	Chloroplast
TaFKBP17-1-6D	TraesCS6D02G132400	0.04	21.85	99,667,030	99,66,9069	6D	845	168	10.06	Chloroplast
TaFKBP16-1-6D	TraesCS6D02G150200	0.19	22.66	123,966,526	1239,688,73	6D	759	208	9.55	Cytoplasmic
TaFKBP16-2-6D	TraesCS6D02G282000	0.13	22.49	389,596,958	389,598,490	6D	1022	222	9.25	Chloroplast
TaFKBP12-6D	TraesCS6D02G293400	0.28	20.45	404,219,105	404,222,466	6D	914	195	9.79	Chloroplast
TaFKBP62-7A	TraesCS7A02G257100	0.65	62.01	246,264,792	246,270,837	7A	2394	559	5.03	Peroxisome
TaFKBP16-3-7A	TraesCS7A02G266500	0.08	24.85	268,865,930	268,867,994	7A	1119	247	7.66	Chloroplast
TaFKBP13-7A	TraesCS7A02G485300	0.06	20.93	676,102,667	676,1039,60	7A	1059	203	8.60	Chloroplast
TaFKBP62-7B	TraesCS7B02G153100	0.65	62.02	203,740,576	203,746,113	7B	2069	559	5.09	Peroxisome
TaFKBP16-3-7B	TraesCS7B02G166000	0.04	21.94	228,914,871	228,916,908	7B	760	220	7.60	Chloroplast
TaFKBP13-7B	TraesCS7B02G388800	0.09	20.86	655,040,607	655,041,893	7B	1065	203	8.37	Chloroplast
TaFKBP62-7D	TraesCS7D02G257300	0.65	61.98	232,811,431	232,816,797	7D	2334	559	5.03	Peroxisome
TaFKBP16-3-7D	TraesCS7D02G268300	0.07	23.76	252,238,804	25,224,0730	7D	1060	235	6.47	Chloroplast
TaFKBP13-7D	TraesCS7D02G472600	0.07	20.9	585,650,878	585,652,169	7D	1057	203	8.60	Chloroplast
TaFKBP18-U	TraesCSU02G108900	0.34	23.59	94,822,583	94,826,891	Un	802	219	10.06	Nuclear, Mitochondrial

GRAVY: grand average of hydropathy index; CDS: coding sequence; PI: protein isoelectric.

**Table 2:** Number of FKBP proteins in different plant species.

Plant Species	Genome Size (Approx.)	Coding Genes	FKBP Genes
*Triticum aestivum* (6n)	17 Gb	107,891	64
*Arabidopsis thaliana* (2n)	135 MB	27,655	23
*Oryza sativa* (2n)	500 MB	37,960	29
*Zea mays* (2n)	2.4 Gb	39,591	30
*Chlamydomonas* (n)	111 MB	17,741	23

## Data Availability

Data are available in the manuscript and in the Supplementary Materials.

## Supplementary Materials

The following supporting information can be downloaded at: https://www.mdpi.com/article/10.3390/ijms232314501/s1.
