# Supplementary material for: Genome-Wide Identification and Analysis of FKBP Gene Family in Wheat (Triticum asetivum)
Source: Int J Mol Sci. 2022 Nov 22;23(23):14501. doi: 10.3390/ijms232314501 (PMC9739119; doi:10.3390/ijms232314501)
Supplement: Supplementary file 1 [file ijms-23-14501-s001.zip › ijms-2004886-supplementary/supplementary-11-2.docx]

**Supplementary Figure**


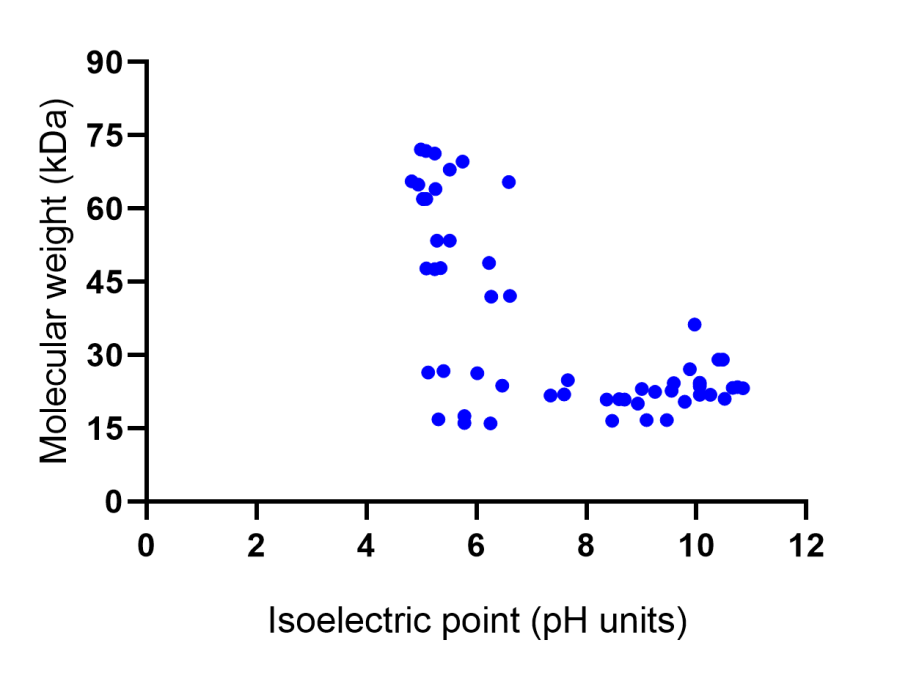


Figure S1. Molecular weight (kDa) vs. isoelectric point plots of TaFKBP proteins. The round shape colors represent the TaFKBP gene family members.


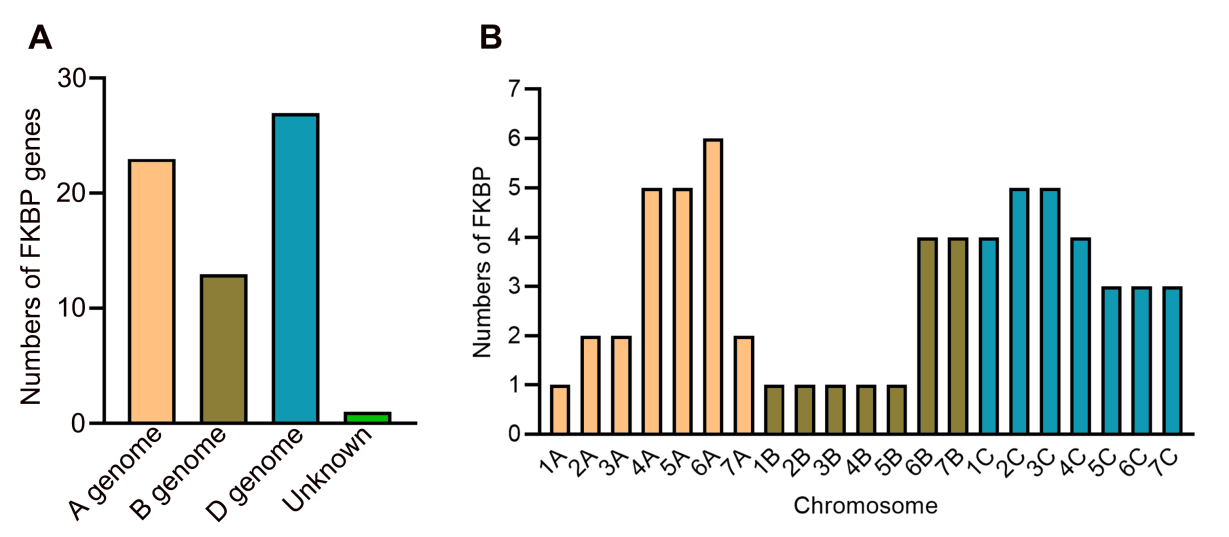


Figure S2. The distribution of FKBP in each sub-genome and chromosome.

1. The distribution of FKBP in A, B and D sub-genome or (B) chromosome.


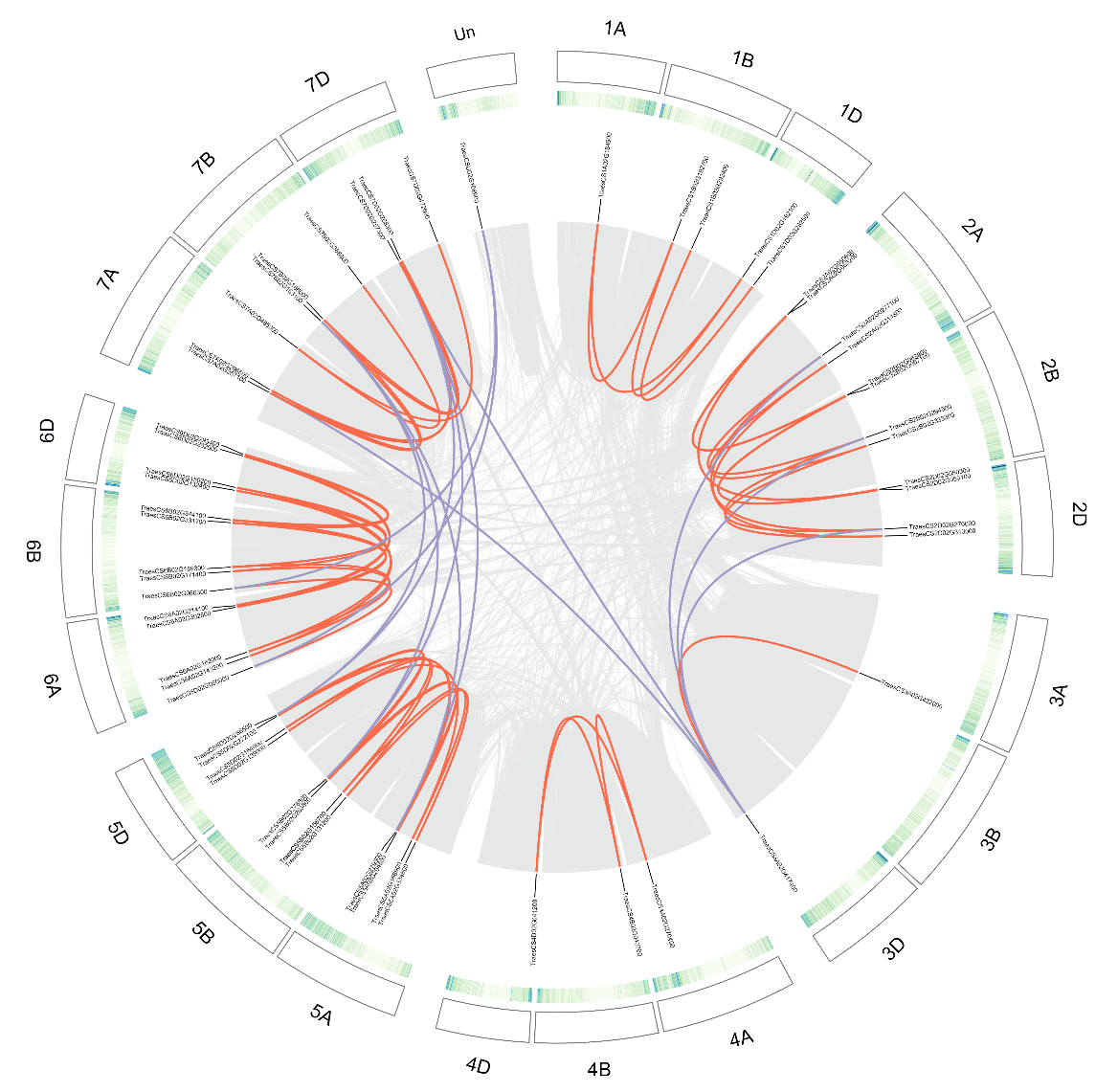


Figure S3. Duplication of the TaFKBP family genes on wheat chromosomes. The gray lines in the background represent the collinear blocks with *Triticum aestivum* genomes, while red and purple line highlight the syntenic FKBP gene pairs.


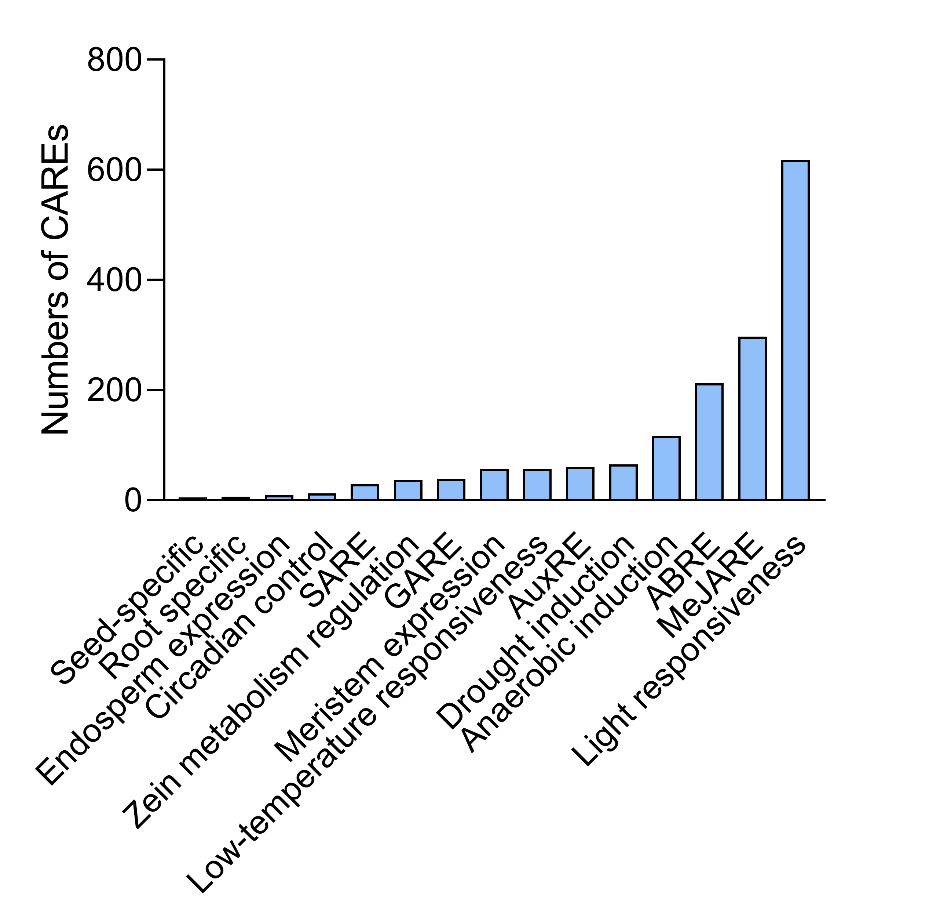


Figure S4. The total of each CAREs in FKBPs promoter regions.

**A**


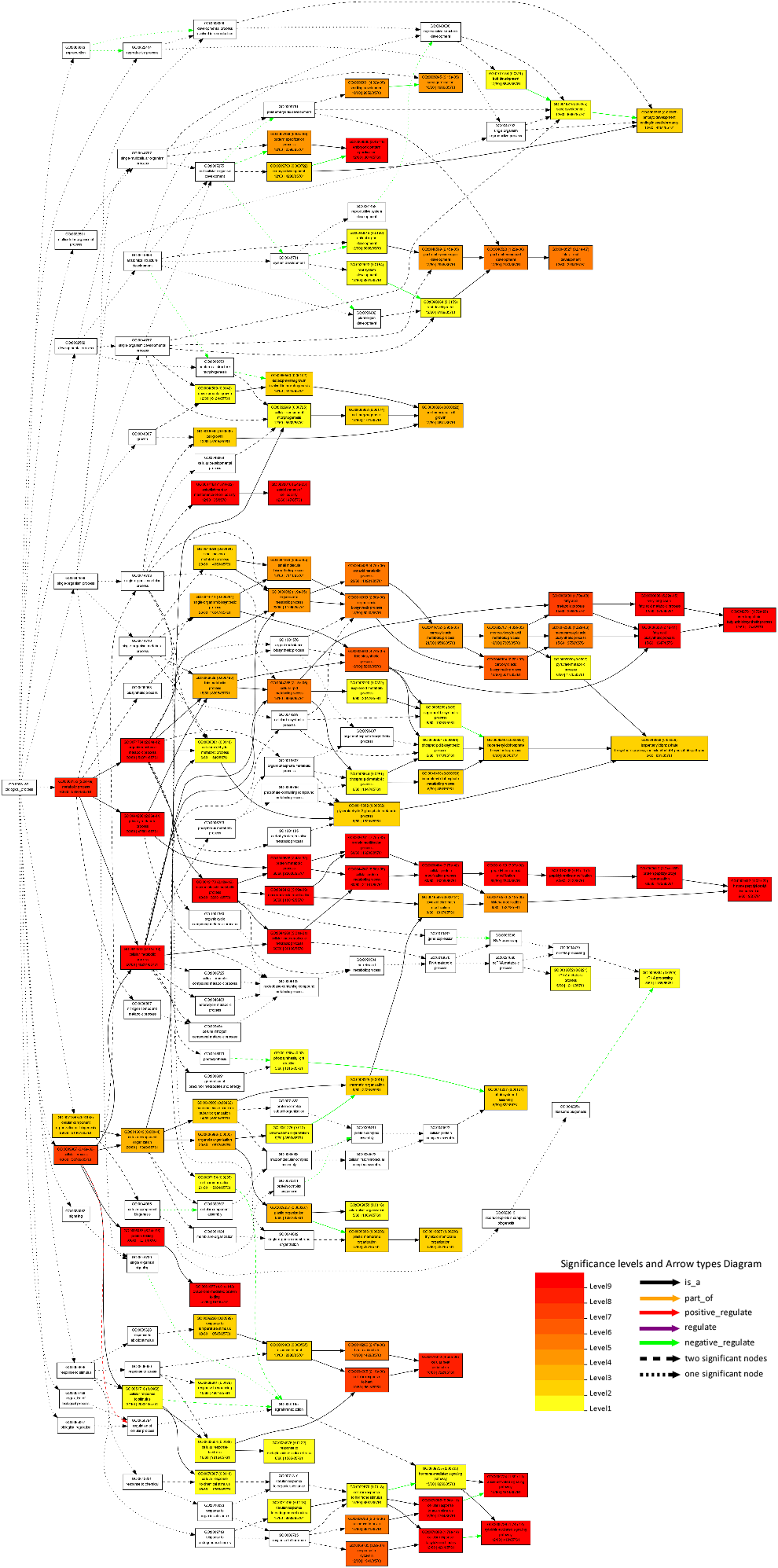


**B**


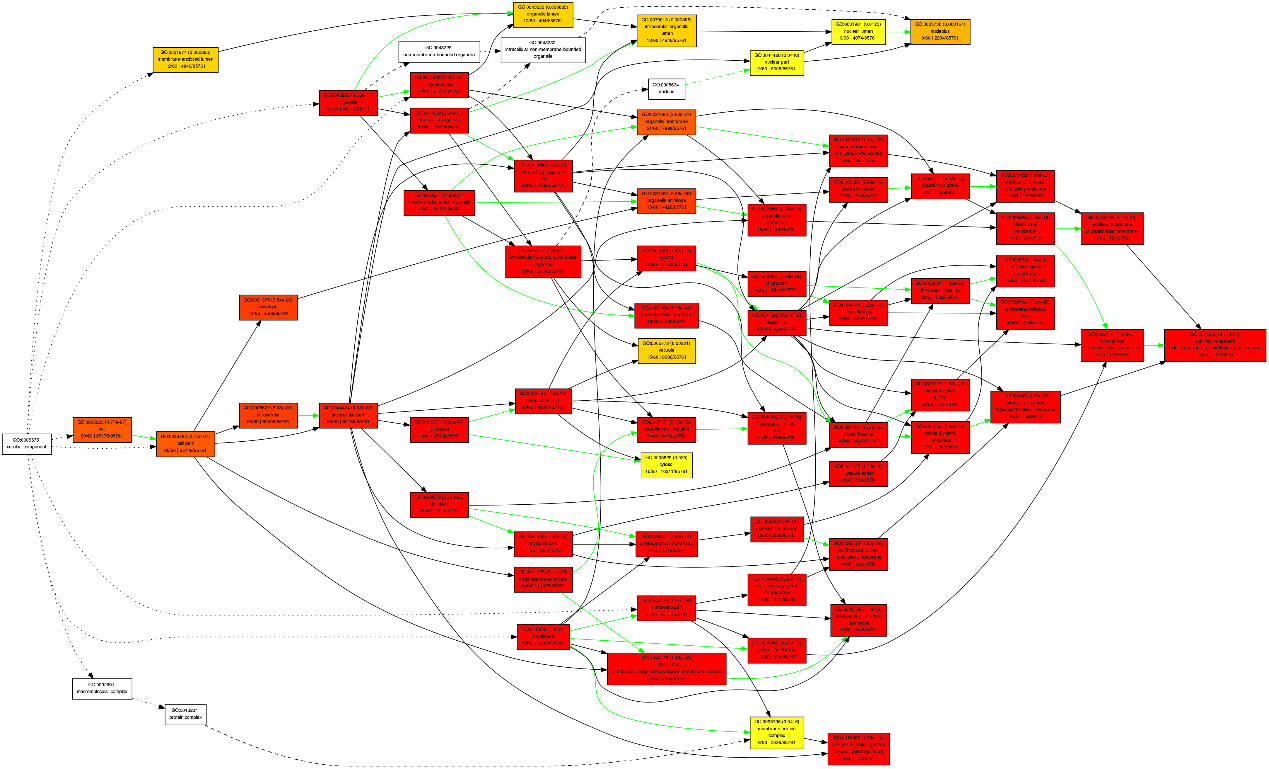


**C**
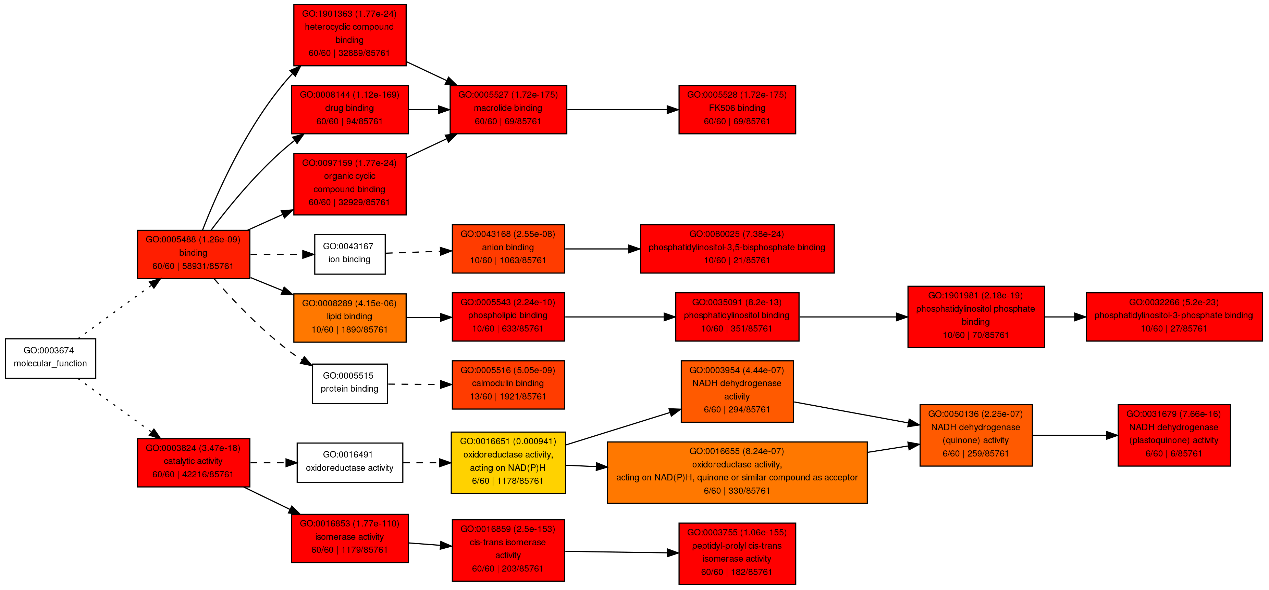


Figure S5. Gene ontology term distribution of TaFKBP gene family predicted using STRING. A. Biological process. B. Cellular component. C. Molecular function.
